# Knowledge and attitude of ICU nurses, students and patients towards the Austrian organ donation law

**DOI:** 10.1186/1472-6939-14-32

**Published:** 2013-08-16

**Authors:** Vanessa Stadlbauer, Peter Steiner, Martin Schweiger, Michael Sereinigg, Karl-Heinz Tscheliessnigg, Wolfgang Freidl, Philipp Stiegler

**Affiliations:** 1 Department of Internal Medicine, Division of Gastroenterology and Hepatology, Medical University of Graz, Graz, Austria; 2 eHealth Master Course, University of Applied Sciences FH Joanneum, Graz, Austria; 3 Institute of Social Medicine and Epidemiology, Medical University of Graz, Graz, Austria; 4 Department of Transplantation Surgery, Medical University of Graz, Graz, Austria; 5 Department for Surgery, Division for Transplant Surgery, Medical University Graz, Auenbruggerplatz 29,A-8036, Graz, Austria

**Keywords:** Organ donation, Legislation, Knowledge and attitude, ICU nurses, Students, Patients

## Abstract

**Background:**

A survey on the knowledge and attitudes towards the Austrian organ donation legislation (an opt-out solution) of selected groups of the Austrian population taking into account factors such as age, gender, level of education, affiliation to healthcare professions and health related studies was conducted.

**Methods:**

An online survey among 3 target groups (ICU nurses, health science students and non health science students) was performed and results were compared to the answers from transplantation patients to a paper questionnaire. A total of 8415 persons were asked to participate in the survey and 2025 (24%) persons correctly completed the questionnaire. 1945 online responses (ICU nurses n = 185; students of health sciences n = 1277; students of non-health science related courses n = 483) were analysed and data were compared to 80 manually filled-in responses from patients from a previous study.

**Results:**

84% of participants state that they know the Austrian organ donation legislation; this percentage varies significantly (p < 0.05) within the target groups and is influenced by demographic variables of the participants. 74% think that the law is good and 79% do not favour a change. Opinions and attitudes towards the legal situation are positively influenced by the affiliation to healthcare professions and health-related fields of study. Interviewed persons who were aware of the legislation before the survey had a more positive attitude towards the existing legislation (77% versus 74%, p < 0.05).

**Conclusions:**

The information level on Austrian organ donation legislation is high. ICU nurses and those who did not know the law before were most critical towards the existing legislation. Therefore education to increase knowledge in the general population and goal-oriented efforts to increase awareness in the target groups should be emphasized.

## Background

Organ transplantation today is the standard therapy of several end-stage diseases. However, the number of patients on the waiting lists exceeds the number of donor organs. In Austria mortality on the waiting list ranged from 2% (pancreas) to 17% (liver) in 2010 and therefore remains unacceptably high even in a country with high organ donor rates [1]. The majority of organs transplanted originate from brain dead organ donors. A multitude of strategies has been implemented to increase organ donor rates [2]. Besides medical strategies to increase organ donation rates, such as the use of marginal donor organs, living donation–in the case of kidney transplantation in Austria only-split organ transplantation or other types of donors such as donation after cardiac death, also the legal framework plays a major role [2]. In different countries different legislations are in place to regulate organ donation from brain dead organ donors. In Austria, since 1982 the law uses a so-called “opt-out” solution for organ donation. A person who does not want to be an organ donor in the case of brain death has to declare this whish before death, for example by putting his/her name on the contradiction registry [3]. About 0.25% of the Austrian population are registered in the contradiction registry. In some European countries the organ donation legislation is different. They have the so-called “opt-in” solution (see Table 1), where potential organ donors have to put their names into a donor registry or to keep their organ donation cards with them and in case of a missing consent of the deceased person the closest relatives (“*next of kin*”) are asked for their agreement [4,5].

**Table 1:** Legislation on organ donation in Europe

**Country**	**Legislation**	**Country**	**Legislation**
Austria	Opt-out	Latvia	Opt-out
Belgium	Opt-out	Lithuania	Opt-in
Bulgaria	Opt-out	Luxembourg	Opt-out
Croatia	Opt-out	Norway	Opt-out
Czech Rep	Opt-out	Portugal	Opt-out
Denmark	Opt-in	Romania	Opt-in
Estonia	Opt-out	Slovenia	Opt-out
Finland	Opt-out	Slovakia	Opt-out
France	Opt-out	Spain	Opt-out
Germany	Opt-in	Sweden	Opt-out
Greece	Opt-out	The Netherlands	Opt-in
Hungary	Opt-out	Turkey	Opt-out
Ireland	Opt-out	UK	Opt-in
Italy	Opt-out	Cyprus	Opt-out

Adapted from [4,5].

The legislation in Austria may be one of the reasons for the relatively high number of organ donors which makes waiting times shorter than in other countries, but still the organ donation rate has to be increased.

For a successful organ donation process it is important that the public and health care professionals have a favourable opinion on the legislation. The need for establishing favourable opinions should not exclude the opportunity for each individual to receive meaningful information that would allow for autonomous decision-making. To promote favourable opinions on the “opt-out” law, we conducted a survey where ICU nurses and students from 2 universities studying in health related and non-health related courses were asked anonymously to donate their opinions on the legislation. The results were compared to data from patients from the transplantation outpatient clinic, who are directly confronted with the problem of organ shortage because they are on the waiting list for whole organ transplantation or have been already transplanted and experienced the waiting-time for receiving an organ often suffering from despair as well as concerns about receiving an organ at all. These data were obtained during a previous study independent of the online survey. The aims of the study were to determine the knowledge about the law and the attitudes towards the Austrian legislation. Factors such as age, gender, level of education, affiliation to healthcare professions and health-related studies were analyzed as well as changes in attitudes depending on the information level in order to get insights what might be helpful to increase the organ donation rate even in a country such as Austria with an opt-out system.

## Methods

### Study group

Between February 2012 and April 2012 a closed online survey was open to responses for 6 weeks. Invitation emails to participate in this survey were sent to the email accounts at university or at work to 3 target groups, to all registered students (n = 3580) at the University of Applied Sciences FH Joanneum in Styria (county in the southeast of Austria), to all registered students (n = 4166) at the Medical University Graz (federal capital of Styria) and to all ICU nurses of all intensive care units at the University Hospital Graz potentially being in charge of organ donors (n = 585). Participants were invited to fill in an 8 questions online questionnaire. Four weeks after the first email a reminder email was sent to the potential participants of the survey to increase the final completeness rates. The results of this survey were compared to a data from a larger survey performed in 2007 in the transplantation outpatient clinic at the Department of Surgery at the Medical University Graz. Patients on the waiting list and transplanted patients (n = 84) were provided with an information leaflet and asked to fill in a paper questionnaire containing the same questions on demographic variables as in the online survey and 2 questions on the knowledge and opinion towards the organ donation law (see Additional file 1). This questionnaire was part of a larger survey on different areas of transplantation and the analysis of other parts (on xenotransplantation) of this questionnaire were already published [6].

The studies were approved by the institutional review board of the Medical University of Graz (EK 24–140 ex 11/12 and EK 18–023 ex 06/07). Participants of the online survey were informed about the purpose, the investigators, the length of the survey and which data were stored. By participating in the survey, informed consent for the data analysis was given. Data storage was carried out anonymously. Patients were included after giving informed consent and could deny participation without giving any reason.

### Questionnaire

The questionnaire which consisted of three pages was created with Freeware Kwik Surveys v47.0. Usability has been tested and technical functionality inclusive checks for consistency and completeness have been performed before fielding the questionnaire. Respondents were able to change their answers by going back to the previous page. A summary of the responses was not given.

Potential participants received an invitation email with a link to the open survey, a web site not used for any other reason. Due to the infrastructure it was not possible to prevent multiple entries from the same individual by checking the IP address or using cookies, as the online survey was supposed to be started within the same network or on the same computer by different participants. No incentives were offered for completing the survey.

Participants were asked to fill in the questionnaire containing 5 questions about demographic data (sex, age group, education level), profession (health related yes/no) and field of study (health related yes/no). Having answered these questions, the participants received information about the necessity of organ transplantations in general and the organ donation legislation in Austria (see Additional file 1 for details) as an integral part of the online survey or as information leaflet. After having read the information, the participants were asked if they already knew the Austrian law before having read the information part of the survey. The following 2 questions focused on the opinions and attitudes towards the law and on the changes of their attitude after having received detailed information about the law. The question measuring opinions and attitudes gave 4 possibilities where the participants were asked to pick either one or more most adequate answers presenting their opinion. The final “shift of opinion” question offered 4 possibilities with single choice to answer. The announcement of this voluntary survey, the questionnaire and the information given to the participants are given as Additional file 1.

After having answered all mandatory questions the <Save>button had to be pressed and data were captured automatically and stored on a secure server by the software. The number of respondents who had started the survey and the number of interviewees who had finally pressed the<Save>button was counted by the application. When the survey had been closed for responses, data were exported as .csv file and imported into the statistics application. Incomplete data sets recognized by missing end time stamps and/or duration time were excluded from analysis.

### Statistics

Descriptive data are presented as percentages of the respective groups. Differences between groups were assessed by two-sided chi-square test. A p < 0.05 was considered as statistically significant. SPSS V20 was used for analysis.

## Results

### Demographic data

**Figure 1:**
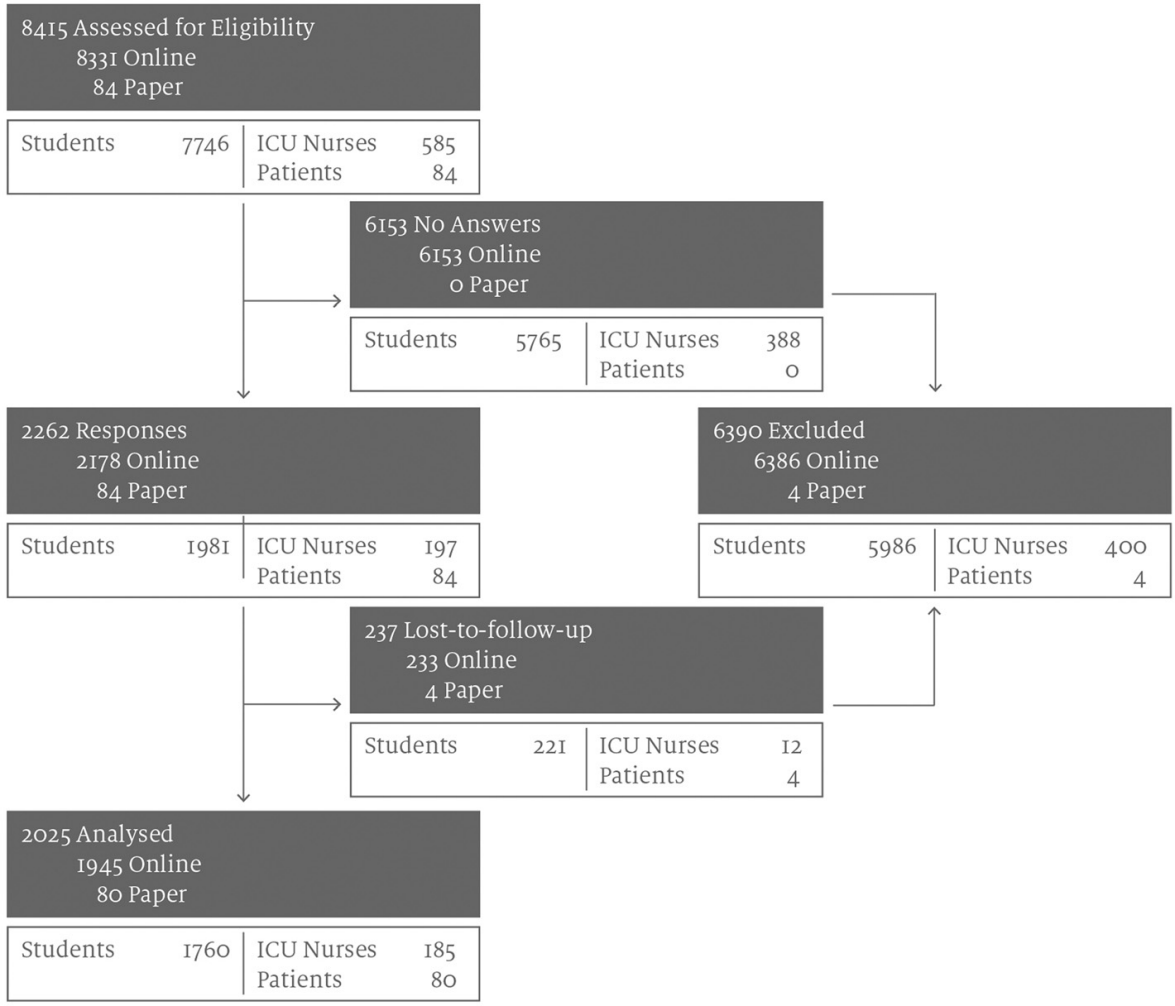
Study flow chart.

The invitation email was sent to 8331 email addresses and in a separate study 84 patients from the transplantation outpatient clinic were invited to fill in the paper questionnaire on an outpatient basis. Overall, 24% of the invited participants completed the survey. Response rate for the online survey was 26% and completion rate 23%. For the paper survey response rate was 100% and completion rate 95%. The final study cohort consisted of 2025 persons (1945 online participants, 80 patients; 65% female, mean age group 26–30) (Figure 1). 61% of participants were between 18 and 25 years old, 21% between 26 and 30 years, 9% between 31 and 40 and 4% between 41 and 50 years and 4% older than 50 years. Education level can be compiled as following: 1% completed primary education, 3% had primary education with an apprenticeship, further 9% had completed secondary education and the remaining 88% had completed higher education, 42% a general and 17% a profession-related high school, 2% an academy and 27% were holding a university degree.

For further analysis the whole cohort was grouped into four groups: ICU nurses (n = 185, 9% of total; 90% female, n = 166), students of health sciences (n = 1277, 63% of total; 66% female, n = 847), students of non-health science related courses (n = 483, 24% of total; 57% female, n = 276) and patients from the transplantation outpatient clinic (n = 80, 4% of total; 24% female, n = 19). For details of distribution of gender, age and education level see Additional file 1: Table S1.

### Information level on organ donation legislation in Austria

**Figure 2:**
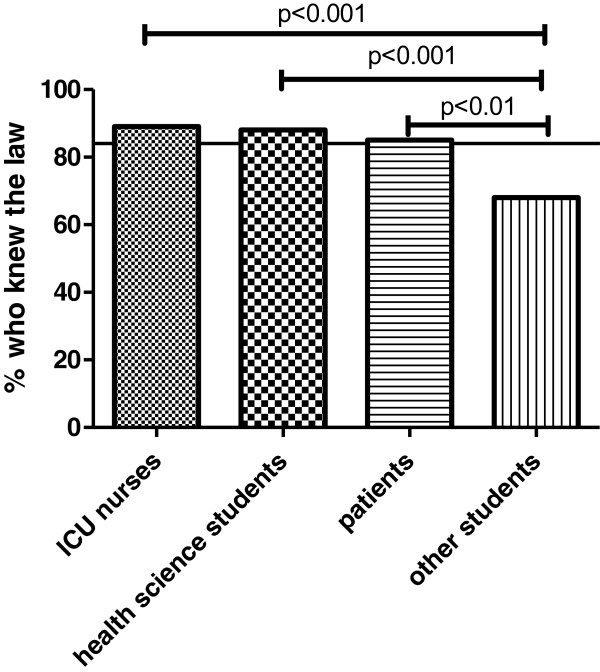
**Percentage of participants by group who stated that they had known the law before.** The black line shows the percentage in the whole study group.

After having read the information page or leaflet, participants of both surveys were asked if they had known the Austrian law on organ donation before. 84% (n = 1692) stated that they had already known it prior to the information provided. Knowledge of the law was significantly higher in the group of ICU nurses (89%, p < 0.05), health science studies (88%, p < 0.05) and patients (85%, p < 0.05) compared to 69% from non-health science related studies. (Figure 2) Age or gender did not influence knowledge about the law significantly. Participants who completed secondary education, had the highest level of knowledge (91%, p < 0.05).

### Attitudes and opinions toward organ donation legislation in Austria

Participants were asked about their attitudes and opinions towards the Austrian law (multiple choice question with multiple answers possible). 74% (1505) are in favour with the Austrian las, 9% (186) thought that the law is ethically not justifiable and that the opt-in solution should be introduced, 44% (884) thought that it is important to consider and accept the opinions of relatives, although the donation rates might decrease, and 29% (591) supported the idea that it should be possible to retrieve organs of potential donors against the will of the relatives in case that the potential donor has not contradicted during lifetime, as the intention of the deceased is not reproducible anymore.

**Figure 3:**
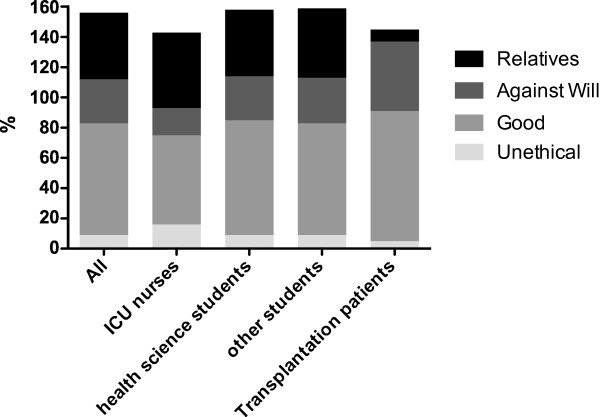
**Compiled the distribution of opinions towards the Austrian organ donation law in the different study groups and in the whole cohort.** Unethical: The law cannot be ethically justified, it is unethical, as every human being should be able to decide by himself, if he or she wants to donate organs or not. An (active) donation register should be introduced. Good: The Austrian legislation is good, as more patients on waiting lists can be cured. Relatives: It is important to consider and accept the opinions of relatives, although the donation rates might decrease. Against will: Provided that potential organ donors did not choose the opt-out option during their lifetimes, it should be possible to retrieve their organs against the will of the relatives, as the intention of the deceased person is not reproducible anymore. Multiple answers were possible for this question, therefore the sum of answers is more than 100%.

16% of ICU nurses thought that the law is unethical compared to 5% of patients (p < 0.05) and compared to 9% of students of health and non health related courses (p < 0.05). On the other hand, only 59% of ICU nurses were of the opinion that the law was good compared to 86% of patients, 76% of students of health sciences and 74% of students of non-health related courses (p < 0.05). 8% of patients thought that it was important to consider and accept the opinions of relatives compared to 50% of ICU nurses, 44% of students of health sciences and 46% of students of non health related courses (p < 0.05). ICU nurses supported the idea that it should be possible to retrieve organs of potential donors also against the will of the relatives to a significantly lower extend (18%) than all other groups (46% of patients, 30% of students of non health related courses and 29% of students of health related courses; p < 0.05) (Figure 3).

Women considered the existing law as unethical in 11%, compared to 6% of men (p < 0.05), women also more frequently thought that it was important to consider and accept the opinions of relatives (47% versus 38%, p < 0.05) and consequently more men supported the idea that it should be possible to retrieve organs of potential donors also against the will of the relatives (37% versus 25%, p < 0.05). The age group with the highest percentage (14%) of participants considering the law unethical were participants aged in between 31 and 40 years. The detailed description of the influence of age, gender and education on the attitude and opinion towards the Austrian donation law is compiled in Additional file 1: Table S2. Due to small sample sizes in some groups, statistical comparison was not performed.

Attitudes and opinions of the participants also depended on the level of knowledge about the law. 8% of participants who stated to know the law considered the law to be unethical compared to 18% of participants who did not know the law (p < 0.05). 77% of participants who stated to know the law considered the law to be good compared to 74% of participants who did not know the law (p < 0.05). 31% of participants who knew the law supported the idea that it should be possible to retrieve organs of potential donors also against the will of the relatives compared to 24% of participants who did not know the law (p < 0.05). There was no difference in the opinion that it was important to consider and accept the opinions of relatives between those who knew or did not know the law prior to reading the information leaflet.

### Wish to change the law and consideration to “opt- out”

Participants of the online survey (n = 1945) were also asked if they would prefer to keep the law as it is or change to the “opt-in” solution or if they definitely or possibly will add their name to the contradiction registry (multiple choice question with single answer possible). 79% (1601) stated that the law should not be changed and that they would not choose the “opt-out” option, 8% (168) favoured the introduction of the “opt-in” option with an active donation registry, 1% (23) definitely wanted to choose the “opt-out” option by being entered in the contradiction registry and 8% (153) stated that they were thinking about being added to the contradiction registry. 4% of participants (transplanted patients and patients on the waiting list for organ transplantation) did not have to answer this question and are therefore missing in the analysis. The detailed description of the influence of age, gender and education on the wish to change the law is compiled in Additional file 1: Table S3.

**Figure 4:**
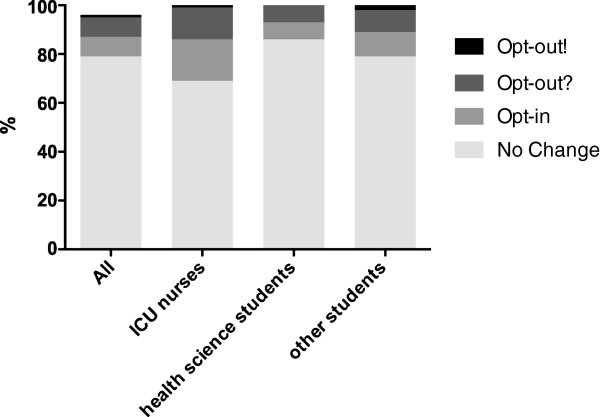
**Wish to change the law and consideration to “opt-out”.** “Opt-out!”: I want to choose the “opt-out” option and definitely plan to be added to the contradiction register. “Opt-out?”: I am thinking about the “opt-out” option. “Opt-in”: I favour the introduction of an (active) donation register, the so called “opt-in” option. No change: The Austrian law should not be changed. 4% of participants (patients) were not asked this question; therefore the sum of answers is less than 100%.

Only 69% of ICU nurses stated that the law should not be changed compared to 86% of students of health sciences (p < 0.05) and to 79% of students of non health related courses (p < 0.05). On the other hand, 17% of ICU nurses favoured the introduction of an “opt-in” registry compared to 7% of students of health sciences (p < 0.05) and to 10% of students of non health related courses (p < 0.05). 13% of ICU nurses stated that they were thinking about being entered in the contradiction registry compared to 7% of students of health sciences (p < 0.05) and 9% of students of non health related courses (p < 0.05). There were no statistically significant differences among the groups for those participants who definitely wanted to choose the “opt-out” option by being entered in the contradiction registry as compiled in Figure 4.

Women were more likely to prefer to switch to the “opt-in” solution whereas men wanted to keep the existing law (p < 0.05). With increasing age significantly more participants voted for the introduction of an “opt-in” system (p < 0.05). Participants having completed secondary education most often voted for the introduction of the “opt-in” system (p < 0.05).

82% of participants who knew the law were against a change in legislation compared to 64% participants who did not know the law (p < 0.05), whereas only 7% participants who knew the law supported the introduction of an “opt-in” register compared to 14% of participants who did not know the law (p < 0.05). 6% of participants who knew the law thought about the possibility to be entered in the contradiction registry compared to 17% of participants who did not know the law (p < 0.05). There was no difference between those who knew the law and those who did not know the law concerning the definite intention to choose the “opt-out” option; details are compiled in Table 2.

**Table 2:** Percentage of participants who knew the law before depending on gender, age and education level in the four groups

**Change in Law**	**All**		**ICU nurses**		**Students health sciences**		**Students non-health sciences**	
	**Law is known**		**Law is known**		**Law is known**		**Law is known**	
	**Y (%)**	**N (%)**	**Y (%)**	**N (%)**	**Y (%)**	**N (%)**	**Y (%)**	**N (%)**
No Change	82	64*	72	50*	88	69*	84	67*
Opt in	7	14*	17	15	6	12*	7	17*
Opt out!	1	1	1	0	1	1	2	2
Opt out?	6	17*	10	35*	5	19*	7	14*

No change: The Austrian law should not be changed and I will not choose the “opt-out” option. Opt-in: I favour the introduction of an (active) donation register, the so called “opt-in” option. “Opt-out”!: I want to choose the “opt-out” option and definitely plan to be added to the contradiction register. “Opt-out”?: I am thinking about the “opt-out” option. * p < 0.05, Chi square test.

## Discussion and conclusion

This survey, containing a short information part on the organ donation law in Austria and 3 questions concerning the law, compared the knowledge about the current organ donation law in Austria and the attitude towards this legislation between ICU nurses, health science students, students of non health science courses. The results of the online survey were compared to data from a paper survey conducted previously with patients from the outpatient transplantation clinic of the Medical University of Graz, who have already received whole organ transplantation or are still on the waiting list for transplantation. The information level on the Austrian organ donation legislation varies in the target groups and is influenced by demographic variables of the participants. Opinions and attitudes towards the legal situation are positively influenced by the affiliation health-related fields of study. Interviewed persons, who had been aware of the legislation before the survey, showed significantly higher agreement with the existing law than those, who reported not to know the law. ICU nurses were the most critical group of interviewees. Our survey was not intended to provide an information intervention and also not designed to reveal the underlying reasons for a critical opinion towards the law; however, from our point of view, the survey may help to intend actions for enlarging the organ donor pool.

The willingness to participate in this online survey was different between the target groups. ICU nurses were most willing to participate in the online survey and female participants were more likely to complete the survey compared to male participants. Because of that and because of the female predominance in some of the target groups, 65% of the participants were women. All patients from the transplantation outpatient clinic, who were asked to participate, were willing to fill in the questionnaire; however, 5% did not complete the questionnaire.

The conduction of online surveys is a valid method to obtain information from a large cohort in a short period of time but also has several limitations: The sample of participants can be biased in a way that only persons interested in the subject will participate. However, being interested in this topic does not mean being supportive towards the organ donation law. In our study 26% of invited subjects participated in the survey, the highest rate of participation was found in the group of ICU nurses, most likely because in this group the personal interest in organ transplantation is highest, since most of the ICU nurses have already had professional experience with the organ donation process. Since we were not able to obtain information if all email-addresses used in the survey were still valid (e.g. because students had graduated), the number of participants might be false low because there might a considerable number of invalid adresses (e.g. because students had graduated). Another possible limitation is that we cannot fully exclude that participants filled in the questionnaire more than once. Since many participants used the same network (university network) or even the same computer in the hospital, we had to allow access by the same IP address more than once. But since there was no reward for participants other than the gained knowledge, we believe that it is unlikely that subjects completed the questionnaire more than once.

Self-reported knowledge of the legislation in Austria was 84% in all groups. Nearly all ICU nurses and students of health care related subjects reported that they knew the law before reading the information page. However, only 59% of students in non health science courses knew the law. Interestingly also 15% of patients in the transplantation outpatient clinic did not know the law. The level of self-reported knowledge in our study however is considerably higher than reported in studies from other countries. Polish theology students only knew the Polish law in 28%, Polish medical students knew the law in only 23%, Swiss first-year medical students in 44% and French first-year medical students in 51% [7-10]. The difference of these studies to our study was that we only asked if the participants knew the law before but we did not verify this self-reported knowledge. There might be a certain amount of participants who think that they know the law but who would in fact not be able to give the correct answer on a question asking if Austria has an “opt-in” or “opt-out” system.

Persons who completed secondary education (e.g. nursing school) were more likely to state that they know the legislation. In other studies young females with higher education have been shown to have the highest knowledge about organ transplantation [11].

We also observed broad agreement with the existing law; out of the cohort who answered the survey 74% were in favor with the Austrian legislation on organ donation. The highest percentage (86%) of agreement with the law was found in the group of patients, while ICU nurses in 16% thought that the law is unethical. Interestingly, also 4 patients from the transplantation outpatient clinic thought that the existing law is unethical. Since the survey was conducted anonymously we were not able to find out the reasons for this opinion or if the patients were already transplanted or still on the waiting list for organ transplantation. Focusing on the results obtained by questioning the ICU nurses, our results are in accordance to literature where ICU nurses have been shown in several studies to be most critical towards the “opt-out” solution in organ donation [8,12,13]. ICU nurses are also reported to have problems in trusting brain death diagnosis [14].

Participants who reported that they knew the law prior to the information which was provided considered the law to be good in a significantly higher proportion than those who stated that they did not know the law before, underpinning the importance of continuous education in the general population and in the target groups who might be involved in organ donation. This notion is also supported by studies from Germany where a strong association between possession of an organ donor card (informed group) and the willingness to donate organs was found [15]. However, a study among students at the University of Regensburg revealed that although nearly 1/3 of the students possess an organ donation card, and therefore could be considered as well informed, there is a considerable lack of knowledge on brain death [16]. However, our study did not directly evaluate the effect of information on the opinion towards the organ donation law.

When we asked the participants if the will of the family should be considered prior to organ donation, about half of the ICU nurses and students but only 8% of patients chose this option. We also asked the participants if they agree that provided that potential organ donors did not choose the “opt-out” option during their lifetimes, it should be possible to retrieve their organs after death against the will of the relatives, as the intention of the deceased person is not reproducible anymore. This scenario, which in theory is covered by the law (in practice the definitive will of relatives will not be overruled), is acceptable for nearly one third of the students but only 18% of the ICU nurses, whereas nearly half of the patients from the transplantation outpatient clinic think that this is acceptable. Patients therefore tended to choose the solution that is most favorable for them in a way that more organs will be available whereas ICU nurses are most concerned about the will of the family, most likely because they are usually in close contact to the family during the process of brain death diagnosis and discussion about organ donation. In an Australian study using a grounded theory approach to elicit community attitudes on deceased organ donation, participants saw a need in a more simple form of family consent, where family members could not overrule the donation decision of the deceased person [17].

The last question of the online survey, which was not included in the paper survey conducted in the transplantation outpatient clinic dealt with the question if participants would like to see a change in the existing law and if they would consider putting their name into the contradiction register. The majority of participants did not wish to change the existing law. However, ICU nurses and those who did not know the law before more often preferred a change to the “opt-in” solution. Also women and those who completed secondary education more often voted for an “opt-in” solution. Since ICU nurses are predominantly female and have completed the nursing school as their highest education, this finding was not unexpected as in general young females with higher education have the highest knowledge on organ transplantation [11].

Adequate and fair communication with the donor families is essential, irrespective of the law in a country [18]. A change from an “opt-in” to an “opt-out” solution for organ donation has been shown to be associated with an increase in organ donation rates. However, when looking at the details of these studies, other factors than presumed consent that had an impact on organ donation rates such as changes in mortality from road traffic accidents and cerebrovascular accident, the development of the transplant programs transplant capacity of a country, economic reasons, religion, education, or public access to information [19]. Several studies from the UK looked at the public support for the change to a presumed consent solution and found that the rate of support increased in the last years (28% to 64%) [19]. A survey among UK faith’s leaders showed that they prefer the “opt-in” solution [20]. In our study we did not include religion as a factor because the vast majority of the Austrian population belongs to a Christian religion and therefore no statistical meaningful conclusions about differences between religious groups could have been drawn.

In summary we could show that the Austrian law on organ donation is well known to ICU nurses, students and patients in the transplantation clinic. There is also broad acceptance of the law. However, especially ICU nurses are more critical and think that the law is unethical. They are also most concerned about the opinion of the relatives of potential organ donors. Only a minor percentage considers putting their name in the Austrian contradiction registry.

These results suggest, that those who are most involved with organ donation in practise are most critical what is in accordance with previous publications [8,12,13]. A questionnaire among ICU staff in Austria revealed that a lack of education and training is one of the key factors for feeling uncomfortable with the process of organ donation [21]. Furthermore healthcare professionals support organ donation in a higher percentage (83%) but more than half of the interviewees wanted to be buried with all their organs intact [22]. This result shows the ambivalence in the opinion of healthcare professionals. The need for education has been shown to be high (21%) in hospital employees [23]. Education is requested by health professionals, can correct false information and might lead to higher organ donation rates [24-26]. However, also legitimate reasons for their concerns, such as problems in accepting the brain death concept or organ procurement procedures might be present. The opportunity to discuss these issues during training programs might be valuable to understand the areas of concern and to develop strategies to overcome these concerns in order to increase the organ donation rate.

In conclusion the widespread support of the existing organ donation law in Austria is encouraging. Targeted education and training programs for health care professionals are warranted. Moreover, a wider range of the general population should be informed about the legislation as the results of our study showed that participants, who are aware of the “opt-out” law, showed a positive attitude towards organ donation. Therefore, educational programs for professionals and public information could be a possibility to increase the support for the current legislation. Our study, however, is not able to answer the question what should be considered education, and what factors other than lack of education could contribute to the critical attitude of ICU nurses towards organ procurement policies and procedures. Therefore further in depth studies on this topic are warranted. However, from our point of view, information about organ donation and organ procurement for the public might be a possibility to increase the organ donation rate in the different countries; independent of the “op-in” or “opt-out” system, whereas we are convinced that an equal legislation in Europe or at least in the EUROTRANSPLANT regions might be helpful to diminish organ shortage and therefore increase organ transplantations and consequently improve patient survival and quality of life.

## Competing interest

The authors declare that they have no competing interests.

## Authors’ contributions

VS designed the study, analysed the data and wrote the paper; PS performed the study, collected and analysed the data and wrote the paper; MS performed the study and collected data, reviewed and discussed the paper; MS performed the study and collected data, reviewed and discussed the paper; KHT designed the study, reviewed and discussed the paper; WF designed the study, reviewed and discussed the paper; PS designed the study, analysed the data and wrote the paper. All authors read and approved the final manuscript.

## Pre-publication history

The pre-publication history for this paper can be accessed here:

http://www.biomedcentral.com/1472-6939/14/32/prepub

## Supplementary Material

Additional file 1 **Online survey announcement.** (translation was performed by the authors, original text in German). **Table S1.** Distribution of gender, age and education level in the four groups in percent per subgroup. **Table S2.** Attitudes and opinions depending on gender, age and education level in the four groups in percent per subgroup. **Table S3.** Reconsiderations depending on gender, age and education level in the three groups from the online survey in percent per subgroup. Click here for file
