# Supplementary material for: Knowledge and attitude of ICU nurses, students and patients towards the Austrian organ donation law
Source: BMC Med Ethics. 2013 Aug 16;14:32. doi: 10.1186/1472-6939-14-32 (PMC3751746; doi:10.1186/1472-6939-14-32)
Supplement: Additional file 1 — Online survey announcement. (translation was performed by the authors, original text in German). Table S1. Distribution of gender, age and education level in the four groups in percent per subgroup. Table S2. Attitudes and opinions depending on gender, age and education level in the four groups in percent per subgroup. Table S3. Reconsiderations depending on gender, age and education level in the three groups from the online survey in percent per subgroup. [file 1472-6939-14-32-S1.docx]

**Supplemental data**

**Online survey announcement**

**(translation was performed by the authors, original text in German)**

We kindly ask you for your support!

Receivers of this announcement to participate in the online survey are persons working in a health-related profession and students who intend to graduate in a health-related field of study or other.

Dr. Stadlbauer-Köllner, head of the research unit “Transplantation Research” at the Medical University in Graz, and DI Peter Steiner, eHealth master student at the University of Applied Sciences in Graz, conduct an online survey on organ donation legislation in Austria. How well known is the law and what are the opinions and attitudes toward it?

**The participation in the survey will not take more than five minutes**. The questionnaire contains some short information about the Austrian law and 8 questions. By participating in the survey, informed consent for the data analysis is given. Please click at the following link to start the survey:

<http://www.kwiksurveys.com?s=OMONGM_e18989ac>

Thank you very much for your participation!

Kind regards,

PD. Dr. Vanessa Stadlbauer-Köllner, DI Peter Steiner

**Transplantation online survey questionnaire**

**(translation by the authors, original questionnaire in German)**

**Demographic data**

1. Gender male

female

1. Age 18 - 25

26 - 30

31 - 40

41 - 50

50+

1. Working as a health care professional? yes

no

1. Do you study health science? yes

no

1. Highest level of education? primary education

primary education with an apprenticeship

secondary education (nursing school)

higher education (general)

higher education (profession-related)

academy (educational, social, …)

university (general, technical, of applied sciences)

**Information on organ donation and legislation in Austria (part of the online survey after the evaluation of demographic data)**

**(translation by the authors, original text in German)**

Currently, there are some diseases that can only be cured by organ transplantation. These diseases can affect the heart (e.g. congenital or acquired cardiomyopathy), the liver (e.g. liver failure, liver cirrhosis), the lung (e.g. lung failure, chronic and obstructive diseases of the lung), the pancreas (e.g. diabetes mellitus that cannot be treated satisfactorily with insulin) or the kidney (e.g. chronic kidney failure).

In Austria, mainly donor organs from ***brain dead patients*** are used. This means that all suitable organs of deceased persons, for example after a car crash, are explanted and given to the most suitable recipient. Therefore, one cannot tell when there will be an organ available for a specific patient on the waiting list. On account of that, surgeries cannot be planned and recipients cannot be prepared for the implantation in an optimal way.

In Austria, we have the so-called ***opt-out solution*** for organ donation. This means that a person who does ***not want*** to be an organ donor in the case of brain death has to put her/his name into a contradiction register. But also the opinion of the patients´ families is respected if they do not want their relative to be an organ donor. In most European countries the organ donation legislation is different. They have the so-called ***opt-in solution***, where potential organ donors have to put their names into a donor registry or to keep their organ donation cards with them. In case of a missing consent of the deceased person the closest relatives (“*next of kin*”) are asked for their agreement. Therefore, in Austria the number of organ donors and the organ donation rates are relatively high which makes waiting times shorter than in other countries. However, there is still a lack of donor organs and a lot of patients die while waiting for an organ.

1. Before you read the information part above, have you been acquainted with the organ donation legislation in Austria?

yes

no

1. What is your opinion and attitude towards the Austrian law (“*multiple choice question with multiple answer*s”)?

The law cannot be ethically justified, it is unethical, as every human being should be able to decide by himself, if he or she wants to donate organs or not. An (active) donation register should be introduced.

Legislation is good, as more patients on waiting lists can be cured.

It is important to consider and accept the opinions of relatives, although the donation rates might decrease.

Provided that potential organ donors did not choose the opt-out option during their lifetimes, it should be possible to explant their organs against the will of the relatives, as the intention of the deceased person is not reproducible anymore.

1. Now, as you have got the information about the donation legislation in Austria, did you change your opinions and attitudes towards the law (“*multiple choice question with single answer*”)?

The Austrian law should not be changed and I will not choose the opt-out option.

I favour the introduction of an (active) donation register, the so called opt-in option.

I want to choose the opt-out option and definitely plan to be added to the contradiction register.

I am thinking about the opt-out option.

Table S1: Distribution of gender, age and education level in the four groups in percent per subgroup

|  | ICU nurses | | | health science students | | | other students | | | transplantation patients | | |
| --- | --- | --- | --- | --- | --- | --- | --- | --- | --- | --- | --- | --- |
|  | All(%) | M(%) | F(%) | All (%) | M(%) | F(%) | All (%) | M(%) | F(%) | All (%) | M(%) | F(%) |
| Gender |  | 10 | 90 |  | 34 | 66 |  | 43 | 57 |  | 76 | 24 |
| Age 18-25 | 20 | 0 | 22 | 72 | 64 | 75 | 60 | 55 | 63 | 0 | 0 | 0 |
| Age 26-30 | 24 | 32 | 23 | 21 | 26 | 18 | 25 | 27 | 24 | 1 | 2 | 0 |
| Age 31-40 | 23 | 37 | 22 | 6 | 8 | 6 | 11 | 14 | 9 | 6 | 8 | 0 |
| Age 41-50 | 22 | 26 | 22 | 1 | 2 | 1 | 4 | 4 | 4 | 14 | 11 | 21 |
| Age 50+ | 11 | 5 | 11 | 0 | 0 | 0 | 0 | 0 | 0 | 79 | 79 | 79 |
| Primary Ed | 1 | 0 | 1 | 1 | 1 | 1 | 0 | 0 | 0 | 11 | 10 | 16 |
| Primary/A | 2 | 5 | 1 | 0 | 0 | 0 | 0 | 1 | 0 | 55 | 54 | 58 |
| Secondary Ed | 75 | 74 | 75 | 1 | 1 | 1 | 2 | 1 | 3 | 11 | 10 | 16 |
| Higher Ed | 22 | 21 | 23 | 98 | 98 | 98 | 98 | 98 | 97 | 23 | 26 | 10 |

Table S2 Attitudes and opinions depending on gender, age and education level in the four groups in percent per subgroup.

|  | ICU nurses | | | health science students | | | other students | | | transplantation patients | | |
| --- | --- | --- | --- | --- | --- | --- | --- | --- | --- | --- | --- | --- |
|  | All (%) | M (%) | F (%) | All (%) | M (%) | F (%) | All (%) | M (%) | F (%) | All (%) | M(%) | F(%) |
| Unethical | 16 | 11 | 16 | 9 | 5 | 10 | 9 | 10 | 9 | 5 | 7 | 0 |
| Good | 59 | 68 | 58 | 76 | 75 | 76 | 74 | 72 | 75 | 86 | 84 | 95 |
| Relatives | 50 | 47 | 50 | 44 | 39 | 46 | 46 | 45 | 47 | 8 | 7 | 11 |
| Against Will | 18 | 21 | 18 | 29 | 38 | 25 | 30 | 34 | 27 | 46 | 46 | 47 |
| Age 18-25 | | | | | | | | | | | | |
| Unethical | 16 |  |  | 8 |  |  | 9 |  |  | 0 |  |  |
| Good | 54 |  |  | 78 |  |  | 75 |  |  | 0 |  |  |
| Relatives | 49 |  |  | 46 |  |  | 47 |  |  | 0 |  |  |
| Against Will | 22 |  |  | 29 |  |  | 30 |  |  | 0 |  |  |
| Age 26-30 | | | | | | | | | | | | |
| Unethical | 16 |  |  | 5 |  |  | 7 |  |  | 0 |  |  |
| Good | 66 |  |  | 75 |  |  | 71 |  |  | 100 |  |  |
| Relatives | 50 |  |  | 40 |  |  | 47 |  |  | 0 |  |  |
| Against Will | 23 |  |  | 31 |  |  | 35 |  |  | 100 |  |  |
| Age 31-40 | | | | | | | | | | | | |
| Unethical | 12 |  |  | 19 |  |  | 9 |  |  | 0 |  |  |
| Good | 56 |  |  | 56 |  |  | 78 |  |  | 100 |  |  |
| Relatives | 63 |  |  | 44 |  |  | 48 |  |  | 0 |  |  |
| Against Will | 14 |  |  | 31 |  |  | 24 |  |  | 20 |  |  |
| Age 41-50 | | | | | | | | | | | | |
| Unethical | 15 |  |  | 24 |  |  | 26 |  |  | 9 |  |  |
| Good | 63 |  |  | 59 |  |  | 68 |  |  | 82 |  |  |
| Relatives | 41 |  |  | 41 |  |  | 26 |  |  | 0 |  |  |
| Against Will | 17 |  |  | 29 |  |  | 16 |  |  | 45 |  |  |
| Age 50+ | | | | | | | | | | | | |
| Unethical | 25 |  |  | 0 |  |  | 0 |  |  | 5 |  |  |
| Good | 50 |  |  | 100 |  |  | 0 |  |  | 86 |  |  |
| Relatives | 40 |  |  | 25 |  |  | 100 |  |  | 10 |  |  |
| Against Will | 10 |  |  | 25 |  |  | 0 |  |  | 48 |  |  |
| Primary education | | | | | | | | | | | | |
| Unethical | 100 |  |  | 14 |  |  | 0 |  |  | 11 |  |  |
| Good | 0 |  |  | 86 |  |  | 0 |  |  | 100 |  |  |
| Relatives | 0 |  |  | 43 |  |  | 0 |  |  | 0 |  |  |
| Against Will | 0 |  |  | 43 |  |  | 0 |  |  | 33 |  |  |
| Primary education with an apprenticeship | | | | | | | | | | | | |
| Unethical | 0 |  |  | 50 |  |  | 50 |  |  | 7 |  |  |
| Good | 100 |  |  | 50 |  |  | 50 |  |  | 80 |  |  |
| Relatives | 33 |  |  | 50 |  |  | 0 |  |  | 7 |  |  |
| Against Will | 0 |  |  | 0 |  |  | 0 |  |  | 43 |  |  |
| Secondary education | | | | | | | | | | | | |
| Unethical | 17 |  |  | 11 |  |  | 11 |  |  | 0 |  |  |
| Good | 59 |  |  | 68 |  |  | 78 |  |  | 89 |  |  |
| Relatives | 52 |  |  | 37 |  |  | 33 |  |  | 11 |  |  |
| Against Will | 17 |  |  | 32 |  |  | 11 |  |  | 67 |  |  |
| Higher education | | | | | | | | | | | | |
| Unethical | 10 |  |  | 8 |  |  | 9 |  |  | 0 |  |  |
| Good | 60 |  |  | 76 |  |  | 74 |  |  | 94 |  |  |
| Relatives | 45 |  |  | 44 |  |  | 47 |  |  | 11 |  |  |
| Against Will | 21 |  |  | 29 |  |  | 31 |  |  | 50 |  |  |

Unethical: The law cannot be ethically justified, it is unethical, as every human being should be able to decide by himself, if he or she wants to donate organs or not. An (active) donation register should be introduced. Good: Legislation is good, as more patients on waiting lists can be cured. Relatives: It is important to consider and accept the opinions of relatives, although the donation rates might decrease. Against will: Provided that potential organ donors did not choose the opt-out option during their lifetimes, it should be possible to retrieve their organs against the will of the relatives, as the intention of the deceased person is not reproducible anymore.

Table S3: Reconsiderations depending on gender, age and education level in the three groups from the online survey in percent per subgroup.

|  | ICU nurses | | | health science students | | | other students | | |
| --- | --- | --- | --- | --- | --- | --- | --- | --- | --- |
|  | All (%) | M (%) | F (%) | All (%) | M (%) | F (%) | All (%) | M (%) | F (%) |
| No Change | 69 | 79 | 68 | 86 | 89 | 84 | 79 | 80 | 78 |
| Opt-In | 17 | 11 | 18 | 7 | 5 | 8 | 10 | 10 | 10 |
| Opt-Out! | 1 | 0 | 1 | 1 | 1 | 1 | 2 | 2 | 2 |
| Opt-Out? | 13 | 11 | 13 | 7 | 5 | 7 | 9 | 8 | 11 |
| Age 18-25 | | | | | | | | | |
| No Change | 60 |  |  | 87 |  |  | 77 |  |  |
| Opt-In | 11 |  |  | 6 |  |  | 10 |  |  |
| Opt-Out! | 0 |  |  | 0 |  |  | 2 |  |  |
| Opt-Out? | 30 |  |  | 7 |  |  | 11 |  |  |
| Age 26-30 | | | | | | | | | |
| No Change | 73 |  |  | 85 |  |  | 83 |  |  |
| Opt-In | 18 |  |  | 7 |  |  | 8 |  |  |
| Opt-Out! | 0 |  |  | 1 |  |  | 2 |  |  |
| Opt-Out? | 9 |  |  | 7 |  |  | 7 |  |  |
| Age 31-40 | | | | | | | | | |
| No Change | 77 |  |  | 75 |  |  | 80 |  |  |
| Opt-In | 14 |  |  | 14 |  |  | 13 |  |  |
| Opt-Out! | 2 |  |  | 5 |  |  | 2 |  |  |
| Opt-Out? | 7 |  |  | 6 |  |  | 6 |  |  |
| Age 41-50 | | | | | | | | | |
| No Change | 81 |  |  | 71 |  |  | 74 |  |  |
| Opt-In | 20 |  |  | 18 |  |  | 16 |  |  |
| Opt-Out! | 0 |  |  | 6 |  |  | 0 |  |  |
| Opt-Out? | 0 |  |  | 8 |  |  | 11 |  |  |
| Age 50+ | | | | | | | | | |
| No Change | 40 |  |  | 75 |  |  | 100 |  |  |
| Opt-In | 25 |  |  | 25 |  |  | 0 |  |  |
| Opt-Out! | 13 |  |  | 0 |  |  | 0 |  |  |
| Opt-Out? | 30 |  |  | 0 |  |  | 0 |  |  |
| Primary education | | | | | | | | | |
| No Change | 0 |  |  | 71 |  |  | 0 |  |  |
| Opt-In | 0 |  |  | 14 |  |  | 0 |  |  |
| Opt-Out! | 0 |  |  | 0 |  |  | 0 |  |  |
| Opt-Out? | 100 |  |  | 14 |  |  | 0 |  |  |
| Primary education with an apprenticeship | | | | | | | | | |
| No Change | 100 |  |  | 50 |  |  | 100 |  |  |
| Opt-In | 0 |  |  | 0 |  |  | 0 |  |  |
| Opt-Out! | 0 |  |  | 0 |  |  | 0 |  |  |
| Opt-Out? | 0 |  |  | 50 |  |  | 0 |  |  |
| Secondary education | | | | | | | | | |
| No Change | 68 |  |  | 84 |  |  | 78 |  |  |
| Opt-In | 17 |  |  | 5 |  |  | 11 |  |  |
| Opt-Out! | 1 |  |  | 5 |  |  | 0 |  |  |
| Opt-Out? | 14 |  |  | 5 |  |  | 11 |  |  |
| Higher education | | | | | | | | | |
| No Change | 74 |  |  | 86 |  |  | 79 |  |  |
| Opt-In | 17 |  |  | 7 |  |  | 10 |  |  |
| Opt-Out! | 0 |  |  | 1 |  |  | 2 |  |  |
| Opt-Out? | 10 |  |  | 6 |  |  | 9 |  |  |

No change: The Austrian law should not be changed and I will not choose the “opt-out” option. “Opt-in”: I favour the introduction of an (active) donation register, the so called “opt-in” option. “Opt-out”!: I want to choose the “opt-out” option and definitely plan to be added to the contradiction register. “Opt-out”?: I am thinking about the “opt-out” option.
